# Effects of Aerobic Exercise on Cardiorespiratory Fitness, Cardiovascular Risk Factors, and Patient-Reported Outcomes in Long-Term Breast Cancer Survivors: Protocol for a Randomized Controlled Trial

**DOI:** 10.2196/45244

**Published:** 2023-03-15

**Authors:** Tormod Skogstad Nilsen, Mali Sæter, Sebastian Imre Sarvari, Kristin Valborg Reinertsen, Sara Hassing Johansen, Elisabeth Rustad Edvardsen, Jostein Hallén, Elisabeth Edvardsen, May Grydeland, Cecilie Essholt Kiserud, Hanne Cathrine Lie, Paul André Solberg, Torbjørn Wisløff, Adam Philip Sharples, Truls Raastad, Kristina Hermann Haugaa, Lene Thorsen

**Affiliations:** 1 Department of Physical Performance The Norwegian School of Sport Sciences Oslo Norway; 2 Institute of Clinical Medicine Faculty of Medicine University of Oslo Oslo Norway; 3 ProCardio Center for Innovation Department of Cardiology Oslo University Hospital Oslo Norway; 4 National Advisory Unit on Late Effects after Cancer Treatment Department of Oncology Oslo University Hospital Oslo Norway; 5 Department of Pulmonary Medicine Oslo University Hospital Oslo Norway; 6 Department of Behavioural Medicine Faculty of Medicine University of Oslo Oslo Norway; 7 Norwegian Olympic and Paralympic Committee and Confederation of Sports Oslo Norway; 8 Health Services Research Unit Akershus University Hospital Lørenskog Norway; 9 Department for Clinical Service Division of Cancer Medicine Oslo University Hospital Oslo Norway

**Keywords:** breast cancer, cardiooncology, cardiorespiratory fitness, exercise medicine

## Abstract

**Background:**

Anthracycline-based chemotherapy has been mainstay of adjuvant breast cancer therapy for decades. Although effective, anthracyclines place long-term breast cancer survivors at risk of late effects, such as reduced cardiorespiratory fitness and increased risk of cardiovascular disease. Previous research has shown beneficial effects of exercise training on cardiorespiratory fitness, but the effects of exercise on limiting factors for cardiorespiratory fitness, cardiovascular risk factors, and patient-reported outcomes in long-term survivors are less clear. Whether previous exposure to breast cancer therapy modulates the effects of exercise is also unknown.

**Objective:**

The primary aim of the CAUSE (Cardiovascular Survivors Exercise) trial is to examine the effect of aerobic exercise on cardiorespiratory fitness in anthracycline-treated long-term breast cancer survivors. Secondary aims are to examine effects of exercise training on limiting factors for cardiorespiratory fitness, cardiovascular risk factors, and patient-reported outcomes, and to compare baseline values and effects of exercise training between similar-aged women with and those without prior breast cancer. A third aim is to examine the 24-month postintervention effects of aerobic exercise on primary and secondary outcomes.

**Methods:**

The CAUSE trial is a 2-armed randomized controlled trial, where 140 long-term breast cancer survivors, 8-12 years post diagnosis, are assigned to a 5-month nonlinear aerobic exercise program with 3 weekly sessions or to standard care. Seventy similar-aged women with no history of cancer will undergo the same exercise program. Cardiorespiratory fitness measured as peak oxygen consumption (VO_2peak_), limiting factors for VO_2peak_ (eg, cardiac function, pulmonary function, hemoglobin mass, blood volume, and skeletal muscle characteristics), cardiovascular risk factors (eg, hypertension, diabetes, dyslipidemia, obesity, physical activity level, and smoking status), and patient-reported outcomes (eg, body image, fatigue, mental health, and health-related quality of life) will be assessed at baseline, post intervention, and 24 months post intervention.

**Results:**

A total of 209 patients were included from October 2020 to August 2022, and postintervention assessments were completed in January 2023. The 24-month follow-up will be completed in February 2025.

**Conclusions:**

The findings from the CAUSE trial will provide novel scientific understanding of the potential benefits of exercise training in long-term breast cancer survivors.

**Trial Registration:**

ClinicalTrials.gov NCT04307407; https://clinicaltrials.gov/ct2/show/NCT04307407

**International Registered Report Identifier (IRRID):**

DERR1-10.2196/45244

## Introduction

### Background

Due to advances in diagnostics and treatment, the 5-year survival rate for early breast cancer has surpassed 90% in the Western world, and the number of breast cancer survivors (BCSs) is steadily increasing. Long-term BCS (ie, living beyond 5 years after diagnosis) are at increased risk of several late effects, such as increased risk of cardiovascular disease (CVD) and incidence cardiovascular (CV) risk factors (eg, obesity, hypertension, and dyslipidemia) [1,2].

Cardiotoxicity is a well-recognized adverse effect of anthracycline therapy, which has been a key component of breast cancer therapy for decades. Anthracycline treatment can induce myocardial damage [3,4] and lead to irreversible left ventricular dysfunction and heart failure [5]. Although the incidence of manifested anthracycline-induced cardiotoxicity after breast cancer treatment is approximately 2%-5% [6,7], the incidence of subclinical left ventricular dysfunction may be significantly higher [8].

Previous studies have demonstrated impaired cardiorespiratory fitness (CRF) in BCSs [9,10]. CRF is an integrative measure of global cardiopulmonary and skeletal muscle function assessed as peak oxygen consumption (VO_2peak_). Reduced CRF is not only an expression of impaired physical functional capacity but is also associated with a greater risk of CVD [11,12]. Cardiac output, pulmonary function, blood oxygen carrying capacity, and peripheral arteriovenous oxygen extraction capacity are physiological determinants of CRF, also termed as limiting factors for CRF. Given the systemic effect of chemotherapy, anthracycline-induced late effects may not be limited to cardiotoxicity, but likely affects the entire cardiopulmonary-muscle axis [13]. In line with this perspective, studies have reported reduced microvessel density, impaired mitochondrial function, and reduced skeletal muscle cell size during anthracycline treatment of breast cancer [14]. To our knowledge, no study has investigated if such adverse effects are present in long-term BCS.

BCS may also face psychosocial late effects, including body image concerns, increased levels of mental distress [15], and chronic fatigue [16,17], potentially negatively affecting their work life and reducing their health-related quality of life (HRQoL) [18,19].

Aerobic exercise has shown to improve CRF in cancer survivors [20], but knowledge on the effects of aerobic exercise on limiting factors for CRF is sparse. To the best of our knowledge, there are no existing data on the effects of exercise training on cardiac morphology and function after anthracycline exposure in long-term cancer survivors.

In nononcological settings, higher levels of physical activity are associated with a dramatic reduction in CVD risk [21]. Studies evaluating such effects among cancer survivors are limited [22]. Adams et al [23] reported improved vascular function and CRF and lower low-density lipoprotein and high-sensitivity C-reactive protein after 12 weeks of supervised high intensity aerobic exercise in survivors of testicular cancer, and similar data on BCS are missing. Finally, exercise training has also been shown to improve mental distress and fatigue [24], and improve HRQoL [25,26] in patients with cancer, but research has mainly been conducted during or shortly after treatment.

### Aims and Hypothesis

The primary aim of this study is to examine the effect of aerobic exercise on CRF in anthracycline-treated long-term BCS compared to standard care. Secondary aims are to examine the effects of exercise training on limiting factors for CRF, CV risk factors, and health-related patient-reported outcomes (PROMs) compared to standard care, and to compare baseline values and effects of exercise training between BCS and similar-aged women without prior cancer. Tertiary aims are to investigate long-term effects of the intervention on CRF, CV risk factors, and health-related outcomes 24 months post intervention. We hypothesize that aerobic exercise will improve CRF.

## Methods

### Ethics Approval

The CAUSE (Cardiovascular Survivors Exercise) trial is conducted according to the Helsinki Declaration, and all study participants will sign informed consent before any study-related procedures. The study is approved by the Regional Committees for Medical and Health Research Ethics (2019/1318) and is preregistered on clinicaltrials.gov (NCT04307407).

### Design

**Figure 1:**
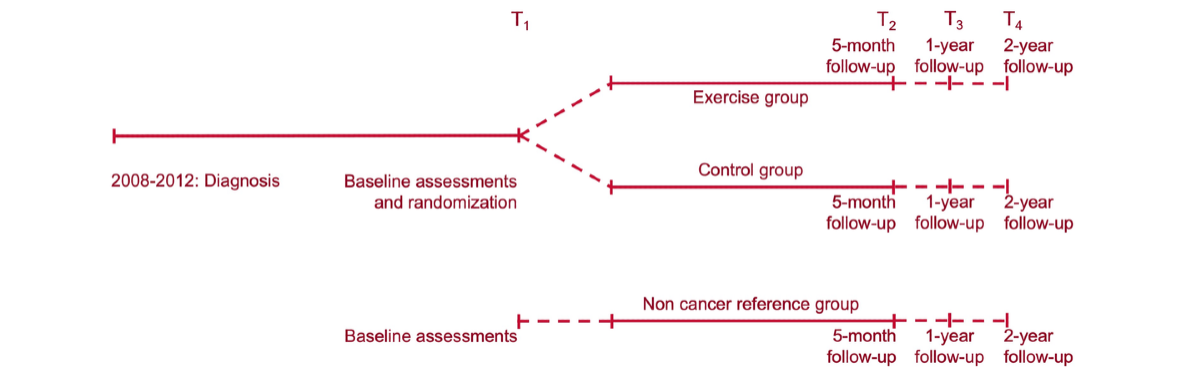
Study outline. Participants in the exercise group, control group, and noncancer reference group undergoes assessments at baseline (T1), after 5 months (post intervention) (T2), and 1 and 2 years post intervention (T3 and T4).

The CAUSE trial is a 2-armed, phase II randomized controlled trial that compares aerobic exercise (exercise group) to standard care (control group) in BCS. A third group comprising similar-aged women with no previous cancer diagnosis is also included (noncancer reference group). The exercise group and the noncancer reference group undergoes the same aerobic exercise program. The overall study design is outlined in Figure 1.

### Participants

#### BCS Group

BCS are invited to participate if diagnosed with human epidermal growth factor receptor 2

–negative breast cancer stage I-III between 2008 and 2012 (ie, 8-14 years after diagnosis), aged 60 years or younger at the time of diagnosis, and received the anthracycline epirubicin as part of their adjuvant therapy. All eligibility criteria are listed in Textbox 1.

**Eligibility criteria for breast cancer survivors in the CAUSE (Cardiovascular Survivors Exercise) trial.**

Inclusion criteria:

- Adjuvant treatment with epirubicin
- Breast cancer diagnosis between 2008 and 2012
- Exercises ≤90 minutes per week
- Physicians’ approval of participation

Exclusion criteria:

- Stage IV breast cancer at diagnosis
- Treatment with trastuzumab
- Recurrence of breast cancer
- Presence of other malignancies (except for basal cell carcinomas)
- Former major cardiac surgery
- Presence of a pacemaker
- Chronic atrial fibrillation
- Recent or uncontrolled cardiovascular disease
- Any physical or mental condition restricting adherence to study procedures (by self-evaluation)

#### Noncancer Reference Group

Participants in the noncancer reference group are women without a history of cancer who otherwise meet the eligibility criteria listed in Textbox 1.

### Recruitment

Women who were diagnosed with breast cancer stage I-III between 2008 and 2012, and live within reasonable distance from Oslo, are identified from the Cancer Registry of Norway. An invitation with study information is sent by mail to all candidates. Thereafter, the study personnel contact the candidates by phone to screen for eligibility and provide additional information. Candidates who meet the eligibility criteria and are willing to participate are then invited to baseline assessments.

Participants for the noncancer reference group are recruited in 2 steps. First, BCS randomized to the exercise group are encouraged to invite a similar-aged friend or relative to participate in the noncancer reference group. The remaining participants in the noncancer reference group are recruited through advertisements in news media and through appropriate social media channels. Interested candidates who meet the eligibility criteria are included.

### Randomization

BCS are block randomized with a 1:1 allocation ratio to the exercise group or to the control group. Due to the strict eligibility criteria, no additional stratification factors are included. A random sequence of study IDs was made by a random number generator prior to inclusion of the first participant. The allocation sequence is concealed from the study personnel involved in baseline assessment until the baseline assessments are completed.

### Assessments

#### Overview

Study outcomes, assessment methods, and assessment times are summarized in Table 1 and described in detail below. Primary outcome is CRF assessed by VO_2peak_. Secondary outcomes are limiting factors for VO_2peak_, CV risk factors, and PROMs. To evaluate long-term effects of the intervention, primary and secondary outcomes are assessed at 24 months (T_4_) postintervention. Some PROMs are also assessed at 12 months (T_3_) postintervention.

All participants are screened by a cardiologist before the assessments. Presence of possible contraindications to exercise training will be evaluated according to the guidelines from the American College of Sports Medicine. [27].

**Table 1:** Outcomes, assessments, and time point of assessments in the CAUSE (Cardiovascular Survivors Exercise) trial.

Domain and outcomes				Assessment method	Time point of assessment
**Primary outcome: cardiorespiratory fitness**					
	Peak oxygen consumption			Treadmill cardiopulmonary exercise test	T_1_, T_2_, T_4_
**Secondary outcomes**					
	**Limiting factors for cardiorespiratory fitness**				
		**Cardiac function**			
			Cardiac dimensions	Echocardiography	T_1_, T_2_, T_4_
			LV^a^ and RV^b^ systolic function	Echocardiography	T_1_, T_2_, T_4_
			LV diastolic function	Echocardiography	T_1_, T_2_, T_4_
			NT-proBNP^c^	Blood sample	T_1_, T_2_, T_4_
		**Blood oxygen transport capacity**			
			Hemoglobin mass	Carbon monoxide rebreathing method	T_1_, T_2_, T_4_
			Hemoglobin concentration and blood volume	Venous blood sample and carbon monoxide rebreathing method	T_1_, T_2_, T_4_
		**Skeletal muscle characteristics**			
			Fiber size	Immunohistochemistry	T_1_, T_2_, T_4_
			Fiber composition	Immunohistochemistry	T_1_, T_2_, T_4_
			Capillaries per fiber	Immunohistochemistry	T_1_, T_2_, T_4_
			Mitochondrial proteins	Western blotting	T_1_, T_2_, T_4_
			Proteins involved in β-oxidation	Western blotting	T_1_, T_2_, T_4_
			Gene expression	Methylome wide arrays	T_1_, T_2_, T_4_
		**Pulmonary function**			
			Forced vital capacity	Spirometry	T_1_, T_2_, T_4_
			Forced expiratory volume in 1 second	Spirometry	T_1_, T_2_, T_4_
			Ventilatory capacity	Estimated from FEV_1_^d^ x 40 or MVV^e^	T_1_, T_2_, T_4_
			DLco^f^ for carbon monoxide	DLco test	T_1_, T_2_, T_4_
			DLco/alveolar volume	DLco test	T_1_, T_2_, T_4_
			Total Lung Capacity-_SB_	DLco test	T_1_, T_2_, T_4_
	**Cardiovascular risk factors**				
		**Hypertension**			
			Blood pressure	Electronic sphygmomanometer	T_1_, T_2_, T_4_
		**Metabolic dysfunction**			
			Fasted glucose, HbA_1c_^g^, insulin	Blood sample	T_1_, T_2_, T_4_
			Lipids	Blood sample	T_1_, T_2_, T_4_
			High-sensitivity C-reactive protein	Blood sample	T_1_, T_2_, T_4_
		**Obesity**			
			BMI	Scale and stadiometer	T_1_, T_2_, T_4_
			Lean body mass	Dual x-ray absorptiometry	T_1_, T_2_, T_4_
			Fat body mass	Dual x-ray absorptiometry	T_1_, T_2_, T_4_
		**Unhealthy lifestyle**			
			Level of physical activity	ActiGraph GT3X+	T_1_, T_2_, T_4_
			Smoking status	HUNT-4^h^	T_1_, T_2_, T_4_
	**Patient-reported outcomes**				
		**Lifestyle**			
			Physical activity and Exercise training	HUNT-4 and GLTEQ^i^ [28]	T_1_, T_2_, T_4_
			Diet	HUNT-4	T_1_, T_2_, T_3_, T_4_
			Sleep habits	Modified HUNT-4	T_1_, T_2_, T_4_^j^
			Work aspects	WAI^k^	T_1_, T_2_, T_4_
		**Health, QoL^l^ and late effects**			
			Comorbidity	Modified HUNT-4	T_1_, T_2_, T_4_
			HRQoL^m^	EORTC^n^ QLQ^o^-C30 [29]	T_1_, T_2_, T_4_
			QoL and Vitality	SWLS^p^ [30] SVS^q^ [31]	T_1_, T_2_, T_4_
			Breast cancer–specific symptoms and complaints^r^	EORTC QLQ-BR23 [32]	T_1_, T_2_, T_4_
			Neuropathy	SCIN^s^ [33]	T_1_, T_2_, T_4_
			Fatigue	Chalder FQ^t^ [34]	T_1_, T_2_, T_4_
			Anxiety Depression symptoms	GAD^u^7 [35] PHQ^v^9 [36]	T_1_, T_2_, T_4_
			Fear of recurrence	ASC^w^ [37]	T_1_, T_2_, T_3_, T_4_
		**Training motivation and mastery**			
			Training motivation	BREQ^x^ 2 [38]	T_1_, T_2_, T_4_^j^
			Exercise competence	PCS^y^ [39]	T_1_, T_2_, T_4_
			Training need satisfaction^z^	BPNES^aa^ [40]	T_1_, T_2_, T_4_^j^

^a^LV: left ventricular.

^b^RV: right ventricular.

^c^NT-proBNP: N-terminal fragment of brain natriuretic peptide.

^d^FEV_1_: forced expiratory volume in 1 second.

^e^MVV: maximal voluntary ventilation.

^f^DLco: diffusion capacity of the lung for carbon monoxide.

^g^HbA_1c_: glycated hemoglobin.

^h^HUNT-4: The Trøndelag Health.

^i^GLTEQ: Godin leisure time exercise questionnaire.

^j^4 and 12 weeks post T_1._

^k^WAI: Work Ability Index.

^l^QoL: quality of life.

^m^HRQoL: health-related quality of life.

^n^EORTC: European Organization for Research and Treatment of Cancer.

^o^QLQ: quality of life questionnaire.

^p^SWLS: satisfaction with life scale.

^q^SVS: subjective vitality scale.

^r^Body image, sexuality, lymphoedema, and pain.

^s^SCIN: scale for chemotherapy-induced long-term neurotoxicity.

^t^FQ: fatigue questionnaire.

^u^GAD: generalized anxiety disorder.

^v^PHQ: patient health questionnaire.

^w^ASC: assessment of survivors concerns.

^x^BREQ-2: Behavioral Regulation in Exercise Questionnaire-2.

^y^PCS: perceived competence scale.

^z^Exercise groups only.

^aa^BPNES: Basic Need Satisfaction in Exercise Scale.

#### Primary Outcome

#### CRF

VO_2peak_ is assessed by an incremental treadmill cardiopulmonary exercise test (CPET) using a modified Balke protocol (RL2700E, Rodby) [41]. The participants are briefly familiarized to treadmill walking prior to the test. The test is continued until voluntary exhaustion despite encouragement by the test leaders. Continuous expired gases are measured breath-by-breath using a gas and volume calibrated metabolic chart (Oxycon Pro, Jaeger GmbH). The highest volume of oxygen consumed across a 30-second period is registered as VO_2peak_. All exercise tests will be supervised by an exercise physiologist and observed by a cardiologist, following standard clinical guidelines [42].

#### Secondary Outcomes

##### Limiting Factors for CRF

The following limiting factors for CRF were will be considered:

- Cardiac function: cardiac morphology and function will be assessed by resting transthoracic echocardiography using Vivid E9 (GE Vingmed). Standard 2D parasternal and apical views are acquired in the end-expiratory phase with participants in the supine left lateral position. Apical 3D recordings will be obtained by stitching together minimum 4 consecutive heart cycles. In addition, left ventricular outflow tract velocity time integral will be obtained immediately after the CPET to estimate maximal stroke volume and cardiac output. Images will be subsequently analyzed offline on EchoPac version 202 (GE Vingmed). Echocardiographic outcomes (listed in Table 1) are measured as recommended by American Society of Echocardiography and European Society of Cardiology [43]. Left ventricular myocardial work will be estimated by a noninvasive method using pressure-strain loop analysis [44].
- Pulmonary function: ventilatory function will be assessed by maximal expiratory flow volume loops (MasterScreen Pneumo Jäger) [45]. The best forced expiratory volume in 1 second and forced vital capacity after 3 acceptable trials will be recorded. Ventilatory capacity, for calculation of breathing reserve during CPET, will be measured by a Maximum Voluntary Ventilation test while breathing as hard and fast as possible for 10 seconds in standing position. Best of 2 maximal attempts will be recorded. The diffusion capacity of the lung for carbon monoxide (DLco) will be measured using the single-breath method, adjusted for serum hemoglobin levels. The average of 2 satisfying attempts will be evaluated for DLco, DLco/alveolar volume (carbon monoxide transfer coefficient), and total lung capacity. All measurements are in accordance with guidelines from The European Respiratory Society/American Thoracic Society [45,46]. The Global Lung Function Initiative reference equations are used, and the outcomes are expressed as absolute values, percent predicted, and *z* scores [47-49].
- Blood oxygen transport capacity: hemoglobin mass will be measured using a carbon monoxide (CO) rebreathing method [50]. First, the participant will be seated for 10 minutes, before capillary blood sampling in two 125-μL preheparinized tubes (Clinitubes; Radiometer). Thereafter, the participant will inhale a bolus of 1 mL/kg body weight of chemically pure CO (AGA Norge) administered via a spirometer (Blood tec GmbH). In a closed circuit, the CO will be rebreathed for 2 minutes together with 3 L of pure oxygen (AGA Norge). Two capillary fingertip blood samples will be collected, 6 and 8 minutes following the administration of CO. All blood samples will be immediately analyzed in duplicate for percent carboxyhemoglobin (ABL80 CO-OX FLEX, Radiometer). Hemoglobin mass will be calculated by dilution of CO in blood with correction for loss of CO to myoglobin (0.3% of the administered CO per minute). Blood volume, plasma volume, and red cell volume will be calculated from hemoglobin mass using venous hemoglobin and venous hematocrit.
- Skeletal muscle characteristics: muscle biopsies will be obtained from vastus lateralis muscle by the Bergström Needle-biopsy technique according to standard procedures established in our laboratory [51]. Muscle fiber size and composition and capillary density will be assessed via immunohistochemistry. The content of mitochondrial proteins and proteins involved in β-oxidation is assessed by western blotting [51]. A subgroup of the muscle biopsies will be processed for RNA and DNA extraction. Methylome wide arrays (eg, 850,000 CpG sites, Infinium Methylation EPIC BeadChip arrays, Illumina, or genome-wide bisulfite sequencing) and transcriptome technology (eg, U133 plus 2.0 array or Claricom S HT Human Array or RNA sequencing) will be applied to assess the expression of epigenetically regulated genes.

##### CV Risk Factors

Resting blood pressure will be measured by an electronic sphygmomanometer (Welch Allyn ProBP 2400) in supine position after echocardiography. Biomarkers listed in Table 1 will be analyzed in accordance with established procedures at Fürst Laboratories from blood samples obtained in a fasted state. Height and weight will be measured by the same scale and stadiometer at all time points (SECA 213). Absolute and relative content of fat mass and lean body mass will be assessed by whole-body dual x-ray absorptiometry (Lunar iDXA, GE Healthcare) in a fasted state. Daily level of physical activity will be measured by accelerometers, using ActiGraph model GT3X+ (ActiGraph LLC). Participants will be instructed to wear accelerometers on the right hip for 7 consecutive days after completion of baseline assessments and during the last week of the intervention period. Smoking status will be recorded.

##### Patient-Reported Outcomes

An overview of the PROMs is presented in Table 1. Sociodemographic variables including marital status, number of children living at home, and education level will be recorded using the same or modified questions from the HUNT-4 (The Trøndelag Health) study [52]. Lifestyle variables include physical activity and exercise measured by questions from HUNT-4 study and Godin Leisure Time Exercise Questionnaire [28], and information on diet, tobacco, alcohol, and sleep habits will be measured by the same or modified questions from the HUNT-4 study. Work aspects will be measured as in the HUNT-4 study and by the Work Ability Index (Finnish Institute of Occupational Health. Work Ability Index, 2006). Information on comorbidities, HRQoL, and general and breast cancer–specific late effects will be measured using a modified version of that used in the HUNT-4 study, the European Organization for Research and Treatment of Cancer Quality of Life Questionnaires (EORTC QLQ C-30 and EORTC QLQ BR-28), including the breast cancer module [29], and the Satisfaction With Life Scale [30,32]. Neuropathy will be assessed by the scale for chemotherapy-induced long-term neurotoxicity [33], fatigue by the Chalder Fatigue Questionnaire [34], vitality by the Subjective Vitality Scale [31], anxiety by Generalized Anxiety Disorder 7 items [35], and depressive symptoms by a modified version of the Patient Health Questionnaire-9 [36]. Fear of recurrence is measured by Assessment of Survivors Concerns questionnaire [37].

Motivation for exercise will be measured by the Behavioral Regulation in Exercise Questionnaire-2 [38] and sense of exercise mastery by Perceived Competence Scale [39]. In the exercise group only, experience with the instructor and exercise program will be measured by the short version of the Health Care Climate Questionnaire [53] and Basic Need Satisfaction in Exercise Scale [40].

### Intervention

**Figure 2:**
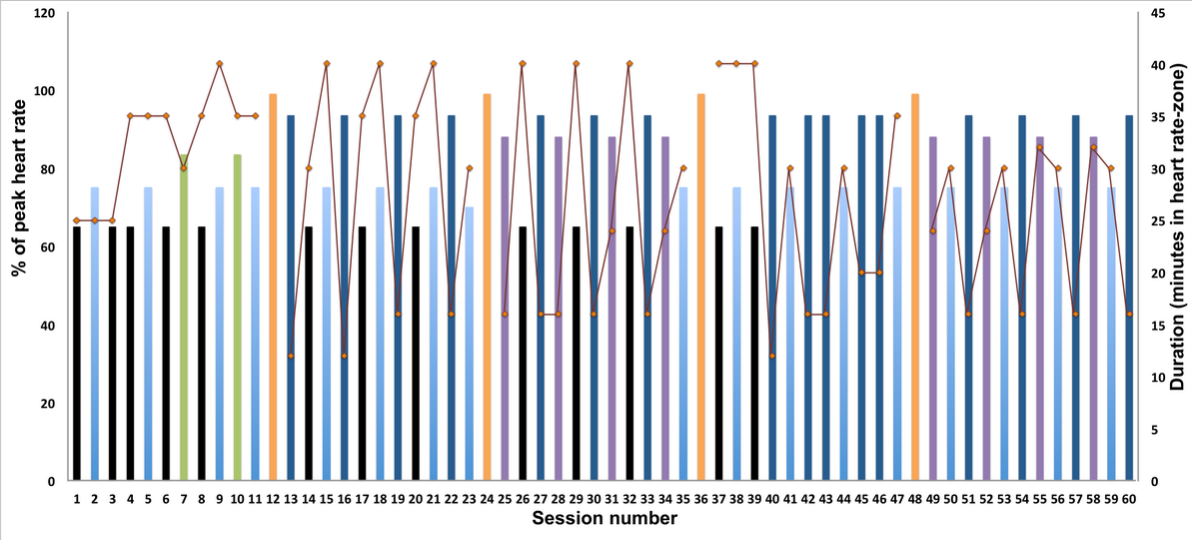
Exercise prescription outline. Bars represents the exercise intensity (% of peak heart rate) at a given exercise session, and the dotted line represents the duration (minutes) at the prescribed exercise intensity in a given session.

The intervention duration is 5 months. Participants in the exercise group perform 3 weekly aerobic exercise sessions aimed at increasing CRF. The exercise plan includes both continuous sessions, with low to moderate exercise intensities, as well as interval sessions performed at higher intensities. The intensity and duration of each individual session and sequencing of the different sessions are outlined in Figure 2. Importantly, the sessions will be scheduled so that the weekly amount of exercise is progressively increased across the intervention period, both in intensity and duration [54]. The preferred exercise modality is inclined treadmill walking or running. The exercise prescription is individually tailored to each participant based on their CPET result.

In Figure 2, the intensity of each session is depicted by the colored bars as a percentage of the mean peak heart rate (HR_peak_) for the session. “Black sessions” are performed at 65% of HR_peak_ for 25-40 minutes (n=13), “light blue sessions” are performed at 75% of HR_peak_ for 30 minutes (n=17), and “green sessions” are performed at 83.5% of HR_peak_ for 30 minutes (n=2). “Dark blue sessions” are performed as three to five 4-minute intervals at 93.5% of HR_peak_ with 3 minutes active rest (brisk walking) between intervals (n=16), and “purple sessions” are performed as two to four 8-minute intervals at 88% of HR_peak_ with 4 minutes active rest (brisk walking) between intervals (n=8). Orange bars represent sessions where the CPET exercise protocol is repeated and are conducted every fourth week (n=4). The treadmill speed and grade at voluntary exhaustion at the CPET protocol sessions will be recorded and used to monitor and report training progression throughout the intervention.

All exercise sessions will be supervised by an instructor at the participant’s local gym. The instructors make sure that the planned exercise sessions are performed and adjust the sessions if necessary. They will also register any deviation from the exercise prescription and report the reason for any adjustment following metrics suggested by Nilsen et al [55]. Adverse events will be reported to the study staff. To increase exercise adherence and educate the instructors, the instructors will be encouraged to undergo a series of web-based seminars. They will be introduced to the rationale for the study, behavior change principles, and how to identify facilitators and barriers to exercise.

BCS randomized to the control group will be encouraged to maintain their current activity level at the time of randomization.

### Statistical Analysis

The sample size calculation was based on the primary outcome VO_2peak_, using a mean change of 3.6 (SD 7.2) mL/kg/minute, from a comparable intervention by Adams et al [23]. With a significance level of 5% and a power of 90% (to compensate for using published results from a single study in our sample size calculation), 63 BCS are needed in each group. To allow for a dropout rate of 7 participants (10%), a total of 70 BCS are included in each group. The same number of participants are included in the reference group. The primary and secondary outcomes are analyzed by generalized linear mixed models. Baseline outcomes in women with and without previous cancer are compared by a 2-sample *t* test.

## Results

The inclusion started in October 2020 and was completed in August 2022. A total of 209 participants were included and postintervention assessments (T_2_) were completed in January 2023. The 24-month follow-up (T_4_) will be completed in February 2025.

## Discussion

Exercise training has previously been shown to improve CRF [20], but there is a paucity of studies investigating the effects of exercise training on other CVD risk factors following cancer treatment [22]. The CAUSE-trial will provide a novel understanding of the potential of exercise training to improve CRF, CV risk profile, and self-reported health-related outcomes in long-term BCS. Since impaired CRF has been reported in cancer survivors [9,10], it is pivotal to understand how previous exposure to cancer treatment modulates the effects of exercise. We hypothesize that previous exposure to cancer treatment, even 10 years ago, would have an impact on the expected results from exercise training. This study will expand on the current literature by thoroughly evaluating exercise effects across the entire cardiopulmonary-muscle axis, and by including an exercise group without a history of cancer. This allows us to directly compare baseline levels and intervention effects between women previously exposed to cancer therapy and unexposed women, and to identify potential bottlenecks to physiological adaptation to exercise training.

As with CRF, the positive effects of exercise training on several PROMs have been reported previously, but the existing literature is mainly limited to effects of exercise training during or shortly after cancer treatment. This is concerning, since approximately 30% of BCS still report chronic fatigue up to 10 years after completing treatment. Results from exercise training performed during or immediately after cancer treatment cannot necessarily be extrapolated to long-term survivors as the mechanisms behind chronic fatigue might differ from the more acute complaints. Although positive effects of exercise training on quality of life already have been demonstrated, less is known about the impact of exercise on other late effects in long-term BCS, such as mental distress, disturbed body image, pain, and sexual health challenges.

One of the major strengths of this study is the solid multidisciplinary constellation of the research group. Our group includes sports physiologists, cardiologists, oncologists, and behavioral scientists. This is also reflected by the list of study outcomes (Table 1) ranging from cardiopulmonary function to skeletal muscle cell function and morphology, and quality of life. Combined with a well-monitored, individually tailored exercise intervention, inclusion of a noncancer reference group, large study cohorts, and longtime follow-up, this will allow for exploring interactions across disciplines.

Our study design has some potential limitations. As with any lifestyle intervention study, we risk recruiting the most motivated and physically active participants, which may compromise the external validity of our results. We aim to reduce this risk of selection bias by recruiting BCS through the Cancer Registry of Norway rather than through media advertisements. Importantly, we use similar exclusion criteria for physical activity levels between cancer survivors and the reference groups, which enable us to identify any modifying effects of previous cancer treatment. Unfortunately, this study lacks completely blinded assessors. As an effort to reduce the influence of known group allocation, assessors will not know the baseline values of participants prior to the postintervention CPET. Furthermore, study staff will use similar feedback and encouragement during all tests. As in all clinical trials, poor intervention adherence and dropouts are major concerns. We attempt to counteract this by using instructors trained in motivational feedback. The knowledge acquired from this study will help to inform future cancer care guidelines.

## Appendix

Multimedia Appendix 1 Peer review report from the Norwegian Cancer Society's Krafttak mot kreft 2018 / Norwegian Cancer Society.

## Abbreviations

BCS: breast cancer survivor
CAUSE: Cardiovascular Survivors Exercise
CO: carbon monoxide
CPET: cardiopulmonary exercise test
CRF: cardiorespiratory fitness
CV: cardiovascular
CVD: cardiovascular disease
DLco: diffusion capacity of the lung for carbon monoxide
HR_peak_: peak heart rate
HRQoL: health-related quality of life
HUNT-4: The Trøndelag Health
PROM: health-related patient-reported outcome
VO_2peak_: peak oxygen consumption

## Data Availability

The data sets generated during and analyzed during the current study are available from the corresponding author on reasonable request.
