# Correction: Effects of Aerobic Exercise on Cardiorespiratory Fitness, Cardiovascular Risk Factors, and Patient-Reported Outcomes in Long-Term Breast Cancer Survivors: Protocol for a Randomized Controlled Trial

**DOI:** 10.2196/54462

**Published:** 2023-11-14

**Authors:** Tormod Skogstad Nilsen, Mali Sæter, Sebastian Imre Sarvari, Kristin Valborg Reinertsen, Sara Hassing Johansen, Elisabeth Rustad Edvardsen, Jostein Hallén, Elisabeth Edvardsen, May Grydeland, Cecilie Essholt Kiserud, Hanne Cathrine Lie, Paul André Solberg, Torbjørn Wisløff, Adam Philip Sharples, Truls Raastad, Kristina Hermann Haugaa, Lene Thorsen

**Affiliations:** 1 Department of Physical Performance The Norwegian School of Sport Sciences Oslo Norway; 2 Institute of Clinical Medicine Faculty of Medicine University of Oslo Oslo Norway; 3 ProCardio Center for Innovation Department of Cardiology Oslo University Hospital Oslo Norway; 4 National Advisory Unit on Late Effects after Cancer Treatment Department of Oncology Oslo University Hospital Oslo Norway; 5 Department of Pulmonary Medicine Oslo University Hospital Oslo Norway; 6 Department of Behavioural Medicine Faculty of Medicine University of Oslo Oslo Norway; 7 Norwegian Olympic and Paralympic Committee and Confederation of Sports Oslo Norway; 8 Health Services Research Unit Akershus University Hospital Lørenskog Norway; 9 Department for Clinical Service Division of Cancer Medicine Oslo University Hospital Oslo Norway

In “Effects of Aerobic Exercise on Cardiorespiratory Fitness, Cardiovascular Risk Factors, and Patient-Reported Outcomes in Long-Term Breast Cancer Survivors: Protocol for a Randomized Controlled Trial” (JMIR Res Protoc 2023;12:e45244) the authors made two corrections.

The affiliations for authors Mali Sæter and Kristina Hermann Haugaawere originally published as:

^2^ ProCardio Center for Innovation, Department of Cardiology, Oslo University Hospital, Oslo, Norway

And have been changed to:

^2^Institute of Clinical Medicine, Faculty of Medicine, University of Oslo, Oslo, Norway

^3^ProCardio Center for Innovation, Department of Cardiology, Oslo University Hospital, Oslo, Norway

The subsequent affiliation numeration has been adjusted to accomodate for this change. The correction will appear in the online version of the paper on the JMIR Publications website on November 15, 2023 together with the publication of this correction notice. Because this was made after submission to PubMed, PubMed Central, and other full-text repositories, the corrected article has also been resubmitted to those repositories.

