# Supplementary material for: Effects of Aerobic Exercise on Cardiorespiratory Fitness, Cardiovascular Risk Factors, and Patient-Reported Outcomes in Long-Term Breast Cancer Survivors: Protocol for a Randomized Controlled Trial
Source: JMIR Res Protoc. 2023 Mar 15;12:e45244. doi: 10.2196/45244 (PMC10131898; doi:10.2196/45244)
Supplement: Multimedia Appendix 1 [file resprot_v12i1e45244_app1.pdf]

NORWEGIAN **CANCER** SOCIETY

Date: 14.12.2018

Dear Tormod Nilsen,

We are pleased to inform you that your grant application to the Norwegian Cancer Society's Krafttak mot kreft 2018 - Late effects of cancer treatment for the research project *The effects of exercise on cardiovascular risk in cancer survivors* has been approved.

The total project grant for the period 01.01.2019 to 31.12.2022 is NOK 5151500.

Please note that if your project receives only partial funding, the Norwegian Cancer Society may request a revised project proposal.

Your application has been carefully reviewed and considered by the peer review committee composed of five international researchers. User representatives have taken part in the assessment process. The aim of user representation is to assess whether user involvement is relevant, and if so, to what degree it is implemented in the project.

An overview of the committee's evaluation of your proposal is provided below:

Relevance to cancer: Ja
Scientific quality: 6
Qualifications of the project manager and project group: 7
Impact: 5
Feasibility: 5

Overall score: 6 (considered fundable > 4.5)

The peer review committee´s comment to your proposal is provided below.

An ambitious and well-written proposal by an early career researcher that has potential to add novel data in the field. The individually tailored intervention supervised by experienced trainers is a strength, as are detailed objective measurements of CVD risk factors and cardiorespiratory fitness. A useful addition to the proposal would have been a discussion around the feasibility to implement the intervention in clinical follow-up practice/recommendations if the trial is successful. User representatives are thoroughly involved in all aspects of the project. The evaluation committee recommends a reduced budget to cover 50% of the salary only for the project manager over the 4 years.

The awarded grant must be accepted by your project administrator. You will receive a copy of the formal contract between Norges Idrettshøgskole and the Norwegian Cancer Society.

Please note that you may not receive funding from two different sources for overlapping projects. In case of funding from both the Norwegian Cancer Society and another source, funding from the other source should be prioritized. In case of funding of two projects from two separate calls from the Norwegian Cancer Society within the same time period, you may only receive one grant.

General information about this year's call will soon be posted on our website. Please note that the right of appeal is limited to case handling errors and misuse of power, and must be received by the Norwegian Cancer Society within three weeks of receiving this letter. We kindly ask that questions regarding the evaluation process and/or the right to appeal be directed to forskningsadministrasjon@kreftforeningen.no.

As a recipient of research funding, we welcome you an award ceremony. The ceremony will take place on Friday January 18 from 11.00-13.00 at the Norwegian Cancer Society's Science Centre, located in Kongens gate 6, Oslo. Please save the date, a more detailed invitation will follow in January.

Sincerely,
The Norwegian Cancer Society

Anne Lise Ryel
Secretary General

Post address: Postboks 4 Sentrum, 0101 Oslo
Visitation address: Kongens gate 6, 0153 Oslo
Telephone: 21 49 49 21
Email: forskningsadministrasjon@kreftforeningen.no
www.kreftforeningen.no

CC: Lise Sofie Woie
